# Circulating levels of monocyte chemoattractant protein‐1 as a potential measure of biological age in mice and frailty in humans

**DOI:** 10.1111/acel.12706

**Published:** 2017-12-31

**Authors:** Matthew J. Yousefzadeh, Marissa J. Schafer, Nicole Noren Hooten, Elizabeth J. Atkinson, Michele K. Evans, Darren J. Baker, Ellen K. Quarles, Paul D. Robbins, Warren C. Ladiges, Nathan K. LeBrasseur, Laura J. Niedernhofer

**Affiliations:** ^1^ Department of Molecular Medicine Center on Aging The Scripps Research Institute Jupiter FL USA; ^2^ Robert and Arlene Kogod Center on Aging Mayo Clinic College of Medicine Rochester MN USA; ^3^ Department of Physical Medicine and Rehabilitation Mayo Clinic College of Medicine Rochester MN USA; ^4^ Laboratory of Epidemiology and Population Science National Institute on Aging National Institutes of Health Baltimore MD USA; ^5^ Division of Biomedical Statistics and Informatics Department of Health Sciences Research Mayo Clinic College of Medicine Rochester MN USA; ^6^ Department of Pediatric and Adolescent Medicine Mayo Clinic College of Medicine Rochester MN USA; ^7^ Department of Pathology University of Washington Seattle WA USA; ^8^ Department of Comparative Medicine University of Washington Seattle WA USA

**Keywords:** biological age, biomarkers of aging, CCL2, chemokine, geropathology, monocyte chemoattractant protein‐1

## Abstract

A serum biomarker of biological versus chronological age would have significant impact on clinical care. It could be used to identify individuals at risk of early‐onset frailty or the multimorbidities associated with old age. It may also serve as a surrogate endpoint in clinical trials targeting mechanisms of aging. Here, we identified MCP‐1/CCL2, a chemokine responsible for recruiting monocytes, as a potential biomarker of biological age. Circulating monocyte chemoattractant protein‐1 (MCP‐1) levels increased in an age‐dependent manner in wild‐type (WT) mice. That age‐dependent increase was accelerated in *Ercc1* ^*−/Δ*^ and *Bubr1* ^*H/H*^ mouse models of progeria. Genetic and pharmacologic interventions that slow aging of *Ercc1* ^*−/Δ*^ and WT mice lowered serum MCP‐1 levels significantly. Finally, in elderly humans with aortic stenosis, MCP‐1 levels were significantly higher in frail individuals compared to nonfrail. These data support the conclusion that MCP‐1 can be used as a measure of mammalian biological age that is responsive to interventions that extend healthy aging.

## 1 INTRODUCTION

Aging is the major risk factor for numerous chronic diseases and is responsible for the bulk of healthcare costs (Goldman et al., 2013). The fastest growing segment of the world population is the elderly, causing an exponential rise in the incidence of chronic diseases. To address this healthcare crisis, there is a growing interest in identifying ways to therapeutically target aging in order to prevent, delay or attenuate multiple age‐related diseases simultaneously (Burd et al., 2016). A number of therapeutic strategies have emerged (Barzilai, Crandall, Kritchevsky & Espeland, 2016; Harrison et al., 2009; Zhu et al., 2015). However, a major barrier to clinical trials targeting aging is the prolonged time between intervention and clinical outcomes (e.g., incidence of age‐related morbidities) and surrogate endpoints are desperately needed. The first clinical trial aimed at delaying the processes that cause aging (TAME: Targeting Aging with Metformin) will soon begin (Barzilai et al., 2016). If this trial is successful, new clinical trials will quickly follow. For these studies, surrogate endpoints will dramatically improve the economy and timescale in which we can measure the effects of interventions on biological age (Niedernhofer, Kirkland & Ladiges, 2016).

Biological age is defined by the health or fitness of an individual, and lack of age‐related diseases, irrespective of their chronological age (Liang et al., 2016). Biological age can be quite distinct from chronological age. For example, cancer survivors are biologically older than their chronological age due to exposure to genotoxic agents, while centenarians are frequently biologically younger than their chronological age (Govindaraju, Atzmon & Barzilai, 2015; Ness et al., 2013). A biomarker of biological age in accessible bodily fluids or tissues would be extremely valuable for clinical trials testing antigeronic factors, but also potentially for triaging patients facing onerous therapeutic procedures. Hundreds of studies have aimed to discover age‐related changes in circulating factors including metabolites, advanced glycation end‐products, exosome content, miRNA, and inflammatory molecules, with varying success. The most successful example of measuring biological age to date is detection of DNA methylation at a subset of CpG islands (Horvath, 2013).

## 2 RESULTS

**Figure 1:**
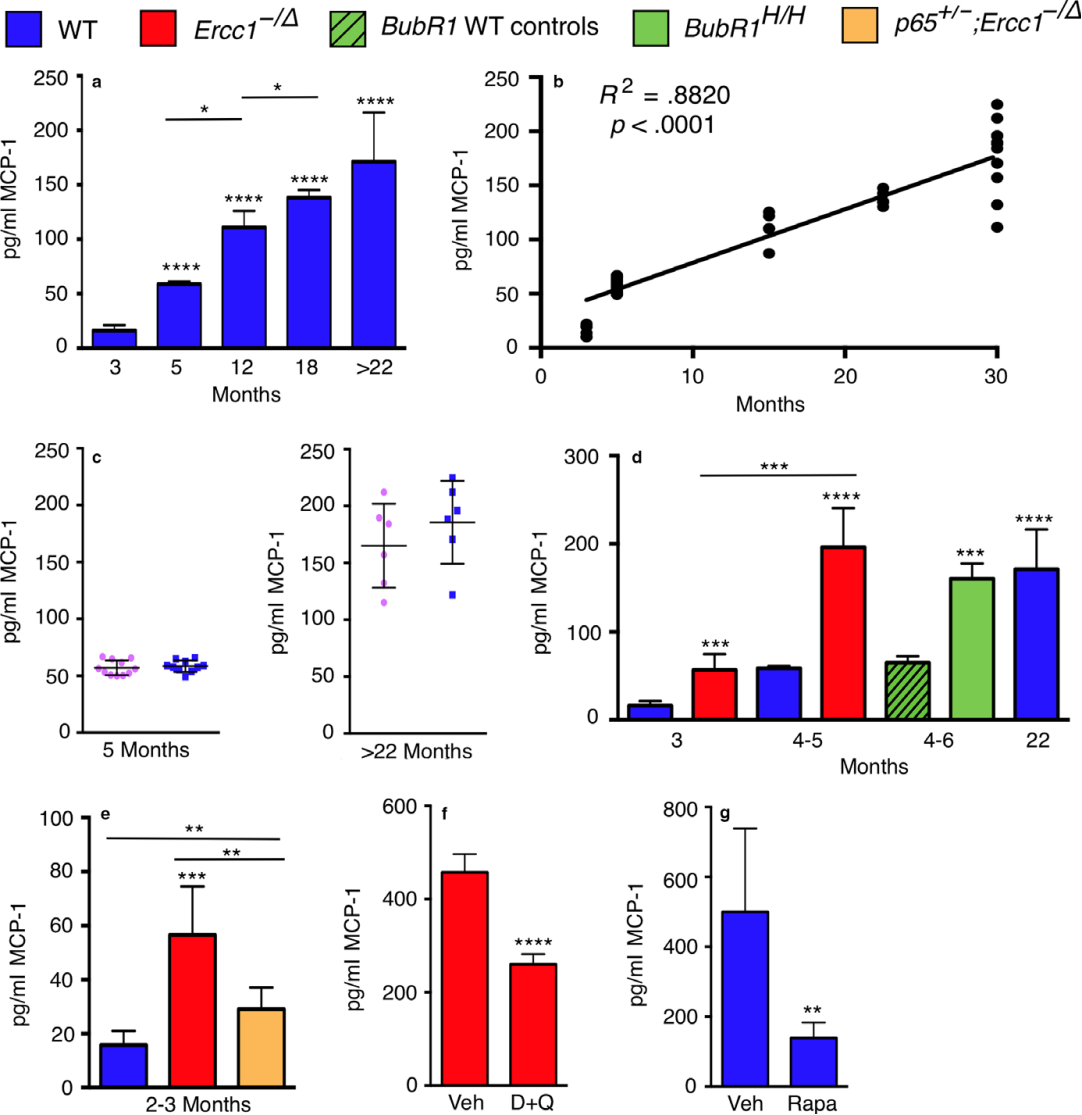
Circulating MCP‐1 levels correlate with biological age. (a) Detection of MCP‐1 in the serum of mice by ELISA. All mice were WT f1 of varying ages and gender. (b) Linear regression analysis of the same data showing a highly significant correlation between serum MCP‐1 and chronological age. (c) Graphing of the same date by gender (pink females; blue male mice). (d) MCP‐1 serum concentrations were quantified by ELISA in progeroid *Ercc1* ^*−/Δ*^ and *Bubr1* ^*H/H*^ mice and WT littermate controls. WT (blue), *Ercc1* ^*−/Δ*^ (red), *Bubr1* ^*H/H*^ (green) and WT controls (green with black slashes). (e) Genetic depletion of NF‐κB in *p65* ^*+/−*^ *;Ercc1* ^*−/Δ*^ mice (yellow), which extends the healthspan of the progeroid mice, reduces MCP‐1 levels relative to *Ercc1* ^*−/Δ*^. Five to six mice were used per group except for Bubr1 and their respective wild‐type controls (n = 3). (f) 16‐week‐old *Ercc1* ^*−/Δ*^ mice (5–6 per group) treated with vehicle (Veh) or a combination of the senolytic drugs dasatinib and quercetin (D+Q) weekly starting at 4–6 weeks, and (g) 26‐month‐old WT mice (6 per group) that were placed on a rapamycin (Rapa) or control (Ctrl) diet for 8 weeks prior to analysis of serum MCP‐1 by ELISA. Values represent the mean ± SD, two‐tailed *t* test. *p* < .05*, *p* < .01**, *p* < .001***, *p* < .0001****, *p* < .00001*****

In hopes of identifying a factor in peripheral blood that correlates with biological age, multiple serum cytokines and chemokines were measured in young and old WT mice using a Luminex platform designed to detect 14 circulating peptides in mouse plasma (Figure S1 and Appendix S1.). Notably, neither TNF‐α nor IL‐6 was increased in aged mice compared to young. In contrast, in this targeted analysis, MCP‐1 was the only peptide that increased significantly and reproducibly with chronological age (Figure 1a). Monocyte chemoattractant protein‐1 (MCP‐1/CCL2) is a chemokine produced by a number of cell types including endothelial, epithelial, mesangial, myocytes, monocytes, and microglial cells, either in a constitutive manner or in response to various stimulants, such as oxidative stress, cytokines, and growth factors (Deshmane, Kremlev, Amini & Sawaya, 2009). Monocyte chemoattractant protein‐1 is a potent monocyte chemoattractant that binds the CCR2 receptor and induces monocytes to exit the bloodstream to become tissue macrophages in response to inflammatory signals (Deshmane et al., 2009).

Numerous studies previously demonstrated that plasma levels of MCP‐1 correlate with chronologic age in humans (Brouwers et al., 2015; Deo et al., 2004; Inadera, Egashira, Takemoto, Ouchi & Matsushima, 1999; Mansfield et al., 2012; Pinke et al., 2013; Scully et al., 2016) and mice (Chiao et al., 2011). Monocyte chemoattractant protein‐1 is a senescence‐associated secretory phenotype (SASP) factor secreted by senescent cells (Jin et al., 2016). Senescence‐associated secretory phenotype can promote secondary senescence in healthy cells (Coppe, Desprez, Krtolica & Campisi, 2010), and senescent cells have been demonstrated to promote aging and age‐related disease (Baker et al., 2011, 2016; Zhu et al., 2015). Circulating levels of MCP‐1 are increased in patients with renal disease (Akdogan et al., 2015), cognitive impairment and Alzheimer's disease (Bettcher et al., 2016), atherosclerosis and cardiovascular disease (Deo et al., 2004). Monocyte chemoattractant protein‐1 is considered to be a marker of “inflammaging,” defined as chronic sterile inflammation that is associated with numerous age‐related diseases (Franceschi & Campisi, 2014). Therefore, we focused on MCP‐1 as a potential biomarker of biological age because it is readily measured in humans, with a relatively small coefficient of variation compared to other inflammatory markers (Figure S2), and there is a rationale for it potentially correlating with aging rather than merely inflammation.

As previously shown in inbred C57BL/6 mice (Chiao et al., 2011), MCP‐1 levels increased linearly with the chronological age of WT f1 mice (FVB/n;C57BL/6; Figure 1b). It is interesting to note that the interindividual variation in MCP‐1 levels increased dramatically in older mice (Figure 1a,b). This is consistent with aging being incredibly heterogeneous at the physiological and molecular level (Burd et al., 2013; Lowsky, Olshansky, Bhattacharya & Goldman, 2014). Also of note, no sex‐based differences in MCP‐1 levels were detected in mice (Figure 1c).

To determine whether MCP‐1 levels corresponded with biological rather than chronological age, we measured serum MCP‐1 in two unrelated models of accelerated aging. *Ercc1* ^*−/Δ*^ mice model a human progeroid syndrome caused by defective DNA repair (Niedernhofer et al., 2006), have a median lifespan of 5 months (Dolle et al., 2011) and spontaneously develop numerous diseases and pathologies associated with old age in humans (Table S1). *BubR1* ^H/H^ mice age rapidly due to defective mitotic spindle assembly checkpoint and have a median lifespan of 6 months (Table S2). In both progeroid strains, serum MCP‐1 levels were significantly increased compared to age‐matched WT mice (Figure 1d). To validate these ELISA data, we used Luminex to measure MCP‐1 in *Ercc1* ^*−/Δ*^ mouse serum and observed a significant increase in MCP‐1 compared to age‐matched controls (Figure S3). Notably, at an age equivalent to the median lifespan of *Ercc1* ^*−/Δ*^ and *BubR1* ^H/H^ mice, serum MCP‐1 levels were equivalent to that of 22‐month‐old WT mice, an age when WT mice begin to display age‐related pathologies (Fox, 2007). The data are not strain dependent as the *Ercc1* ^*−/Δ*^ and naturally aged mice were in an f1 (C57BL/6;FVB) genetic background, while the *BubR1* ^H/H^ mice were C57BL/6.

To determine whether MCP‐1 levels can detect reduced biological age, serum chemokine levels were measured in *p65* ^+/*−*^;*Ercc1* ^*−*/*Δ*^ mice. We previously established that genetic depletion of the RelA/p65 subunit of NF‐κB significantly extends the healthspan of *Ercc1* ^*−/Δ*^ mice (Tilstra et al., 2012). Indeed, *p65* ^+/*−*^;*Ercc1* ^*−*/*Δ*^ mice had significantly reduced circulating levels of MCP‐1 compared to age‐matched *Ercc1* ^*−/Δ*^ mice (Figure 1e). Together, these data support the conclusion that MCP‐1 is a better marker of biological than chronological age.

Monocyte chemoattractant protein‐1 expression is increased in fibroblasts from Hutchinson–Gilford progeria syndrome patients compared to control cell lines (Csoka et al., 2004). This was recapitulated in mouse embryonic fibroblasts derived from *Ercc1*‐deficient mice. Monocyte chemoattractant protein‐1 expression was elevated in *Ercc1* ^*−*/*−*^ MEFs compared to WT as early as passage 2 and levels increased significantly in both WT and *Ercc1* ^*−*/*−*^ cells with passaging (Figure S4a and Table S3 for primers). Similarly, MCP‐1 protein abundance was higher in the media of p7 cells compared to p2, and significantly greater in *Ercc1* ^*−*/*−*^ MEFs compared to WT (Figure S4b). The MCP‐1 data corresponded with a significant increase in the expression of other markers of cellular senescence in the *Ercc1* ^*−*/*−*^ cells relative to WT (*p16* and *p21*; Figure S4c‐d). Thus, MCP‐1 expression, at both the RNA and protein level, may serve as an indicator of the burden of senescent cells, which drive aging.

By definition, a biomarker of biological age should respond to therapeutic interventions proven to significantly improve healthspan or lifespan. Here, we measured serum MCP‐1 in two distinct, established intervention paradigms. Genetic or pharmacologic ablation of senescent cells extends healthspan of mice (Baker et al., 2016; Zhu et al., 2015). A combination of two senolytic drugs (dasatinib and quercetin) extends the healthspan of *Ercc1* ^*−/Δ*^ mice and delays multiple age‐related pathologies (Zhu et al., 2015). In that study, *Ercc1* ^*−/Δ*^ mice were treated weekly with a combination of dasatinib (5 mg/kg) and quercetin (50 mg/kg) for 10 weeks, starting at 6 weeks of age. Here, we analyzed serum from these mice for circulating levels of MCP‐1. *Ercc1* ^*−/Δ*^ mice treated with D+Q had significantly lower circulating concentrations of MCP‐1 than vehicle‐treated controls (Figure 1f). Of note, serum MCP‐1 levels in the vehicle only group of *Ercc1* ^*−/Δ*^ mice in this study are higher than those of untreated animals *Ercc1* ^*−/Δ*^ mice (4–6 months *Ercc1* ^*−/Δ*^ mice in Figure 1d was ~175 pg/mL vs. ~400 pg/ml in 4‐month‐old mice in Figure 1f). We attribute this to the repeated i.p. injections and frequent handling of the *Ercc1* ^*−/Δ*^ mice in the latter study, which exacerbates their frailty.

Rapamycin, an inhibitor of the mTOR kinase, causes a significant extension in the lifespan of WT mice (Harrison et al., 2009). Furthermore, late‐life intervention with rapamycin is sufficient to reduce multiple characteristics of cardiac aging (Dai et al., 2014). Two‐year‐old C57BL/6J mice were fed a diet containing rapamycin (14 ppm for females or 42 ppm for males) or a control diet for 2 months. Longitudinal echocardiography demonstrated that rapamycin significantly reversed aging‐related decline in cardiac performance and substantially attenuated cardiac hypertrophy, as previously described (Dai et al., 2014). In addition, rapamycin attenuated composite lesion scores in kidneys (Figure S5), liver, and lungs of these mice by an average of 40%, 41%, and 29%, respectively. Composite lesion scores generated by a geropathology grading platform have been shown to increase in mice in an age‐dependent manner and align with biological age (Ladiges et al., 2017). Serum levels of MCP‐1 were significantly decreased in 26‐month‐old WT mice after treatment with rapamycin compared to controls (Figure 1g). These data provide strong experimental evidence that in preclinical models, circulating MCP‐1 levels serve as a surrogate endpoint; that is, it responds to interventions that improve clinical endpoints of healthy aging, irrespective of the chronological age of the animals.

Interestingly, MCP‐1 levels were greater in inbred C57BL/6NJ mice compared to age‐matched f1 mice (~500 pg/ml for vehicle‐treated 26‐month‐old C57BL/6NJ mice in Figure 1f compared to ~175 pg/ml for f1 C57BL/6J:FVB/NJ mice >22 months of age in Figure 1a). This suggests that f1 mice are biologically younger than chronologically age‐matched inbred mice. In fact, f1 mice are healthier and longer‐lived than inbred mice (Flurkey, Currer & Harrison, 2006). In addition, inbred mice accumulate numerous age‐related histopathological lesions in multiple organs at an earlier age than f1 mice (Ladiges et al., 2017) (Figure S6). The fact that rapamycin lowers serum MCP‐1 levels to a range consistent with f1 mice suggests that rapamycin *reverses* aging.

**Figure 2:**
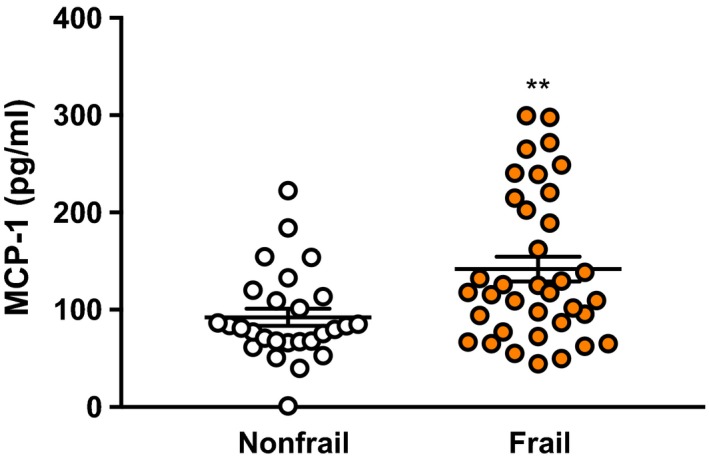
Circulating MCP‐1 levels are elevated in frail older adults. Plasma MCP‐1 concentrations were quantified by a Luminex platform. Frail individuals possessed three or more of the following criteria: slow gait, weak grip, reduced physical activity, low endurance, and unintentional weight loss. Graphed are individual values. The black bars represent the mean ± SEM (nonfrail *n* = 27, frail *n* = 36, Mann–Whitney test, **p = .009)

Our findings demonstrate striking associations between circulating MCP‐1 concentrations and biological age in multiple mouse strains. However, establishing whether a comparable relationship exists in humans is necessary for determining translational utility. Accordingly, we measured plasma MCP‐1 levels in a cohort of older adults undergoing valve replacement surgery for severe aortic stenosis (Table S4). Cardiovascular health study (CHS) frailty testing was conducted as a surrogate measure of biological age, using the presence of three or more frailty criteria (slow gait, weak grip, reduced physical activity, low endurance, and unintentional weight loss) as an operational frailty definition (Fried et al., 2001). Within this sample of 27 women and 36 men, mean age of 81 years, circulating MCP‐1 levels were 54% higher in frail participants (Figure 2). As frailty status was associated with age and sex (Table S4), we also applied linear regression analyses to control for these factors. A one unit increase in the natural log of MCP‐1 levels was associated with a 0.86 unit increase in frailty score, and the strength and significance of this relationship did not meaningfully change after adjusting for age, sex, or combined age and sex (Table S5). To further explore potential sex differences, we split our sample into male and female groups and applied univariate linear regression. A one unit increase in natural log MCP‐1 levels corresponded to a 0.74 and 1.45 unit increase in frailty score in women (*p* = .004) and men (*p *= .002), respectively (Table S6; Figure S7). Thus, we conclude that circulating MCP‐1 concentrations are a robust indicator of biological age in humans, regardless of sex.

## 3 DISCUSSION

There are many prior studies correlating inflammatory biomarkers with chronological age, age‐related disease or functional decline (Charlton et al., 2017; Collerton et al., 2012; Figueroa‐Vega, Moreno‐Frias & Malacara, 2015; Franceschi, Monti, Sansoni & Cossarizza, 1995; Julian et al., 2015; Kleinschmidt et al., 2016; Lippi, Sanchis‐Gomar & Montagnana, 2014; Lu et al., 2016; Matsushima et al., 2015; Nadrowski et al., 2016; Noren Hooten, Ejiogu, Zonderman & Evans, 2012; Sesso et al., 2015). A very comprehensive study by Collerton et al. identified low IL‐6 or TNF‐α as negatively correlating with risk of frailty, while high C‐reactive protein and low albumin correlated with a high risk of frailty (Collerton et al., 2012). MCP‐1 was not measured in this study. In contrast to our study, Lu et al., in a small study, found MCP‐1 to be negatively associated with frailty, as was IL‐6R (Lu et al., 2016). Other studies found a lack of correlation between inflammatory biomarkers and a decline in cognitive function (Julian et al., 2015; Matsushima et al., 2015) or risk of cardiovascular disease (Sesso et al., 2015). In these studies, MCP‐1/CCL2 was not measured.

In summary, we report for the first time that circulating levels of MCP‐1 correlate with biological age of mammals. This is supported by data in both mice and humans. MCP‐1 is a SASP factor secreted by senescent cells (Jin et al., 2016). Senescent cells and the pro‐inflammatory cytokines that they secrete negatively affect tissue homeostasis and repair, leading to organ dysfunction and aging (van Deursen, 2014). Thus, elevated MCP‐1 levels could correlate with increased biological age because it reflects a greater burden of senescent cells and/or a state of sterile inflammation that is known to promote aging and age‐related disease (Tchkonia, Zhu, van Deursen, Campisi & Kirkland, 2013). Because of the urgent need for measures of biological age, further studies are needed to reproduce this study, validate MCP‐1 in other systems, and determine its power to predict morbidity and mortality in prospective studies.

## AUTHOR CONTRIBUTIONS

MJY did the experiments with mouse serum. MJS, EJA and NKL contributed data from the human frailty study. NNH and MKE contributed data from the HANDLS cohort. EKQ provided rapamycin‐treated mouse tissues. DJB contributed *BubR1* ^H/H^ mouse tissues. WCL contributed geropathology assessment. PDR contributed to D+Q and p65 studies. MJY, MJS, NKL, NNH, MKE and LJN did the data analysis and prepared the manuscript.

## CONFLICT OF INTEREST

None declared.
