# Supplementary material for: Circulating levels of monocyte chemoattractant protein‐1 as a potential measure of biological age in mice and frailty in humans
Source: Aging Cell. 2017 Dec 31;17(2):e12706. doi: 10.1111/acel.12706 (PMC5847863; doi:10.1111/acel.12706)
Supplement: Supplementary file 1 [file ACEL-17-e12706-s001.docx]

**Supplemental Figure 1. Multiplex ELISA of serum metabolic hormones and peptides.** Serum from 2-3 month old (blue) and 24 month old (green) WT mice were analyzed for 14 different hormones and peptides using the Milliplex Mouse Metabolic Hormone Panel Kit (5-15 mice per group).

**Supplemental Figure 2. Coefficient of variance for human chemokines and cytokines.** Various chemokines and cytokines were measured in serum from 78 females from the HANDLS study. The coefficient of variance is plotted for each serum peptide.

#

**Supplemental Figure 3. Analysis of circulating MCP-1 in progeroid mice.** MCP-1 was measured in the serum of age-matched WT (blue) and *Ercc1^-/Δ^* (red) mice using the Milliplex Mouse Metabolic Hormone Panel Kit (9-11 mice per group). The values represent the mean ± SD. ****p<0.0001 using a two-tailed Student’s *t* test.

**Supplemental Figure 4. Fibroblasts derived from progeroid mice express elevated levels of *Mcp1*.** (**A**) WT and *Ercc1*^-/-^ primary MEFs were analyzed for expression of *Mcp1* at passage 2 (P2) and 7 (P7) by qPCR. (**B**) Conditioned media was analyzed for MCP-1 expression by ELISA. Senescence markers (**C**) *p16* and (**D**) *p21* expression in cell lysates was also measured by qPCR. Data was analyzed by the ΔΔCt method and expression was normalized to *Gapdh* expression*.* WT (blue) and *Ercc1^-/-^* (red). Values represent the mean ± SD for n=3 independent MEF lines per group. A one-way ANOVA was used for statistical analysis, p<0.05 *, p<0.01 **, p<0.001 ***, p<0.0001 ****

**A**


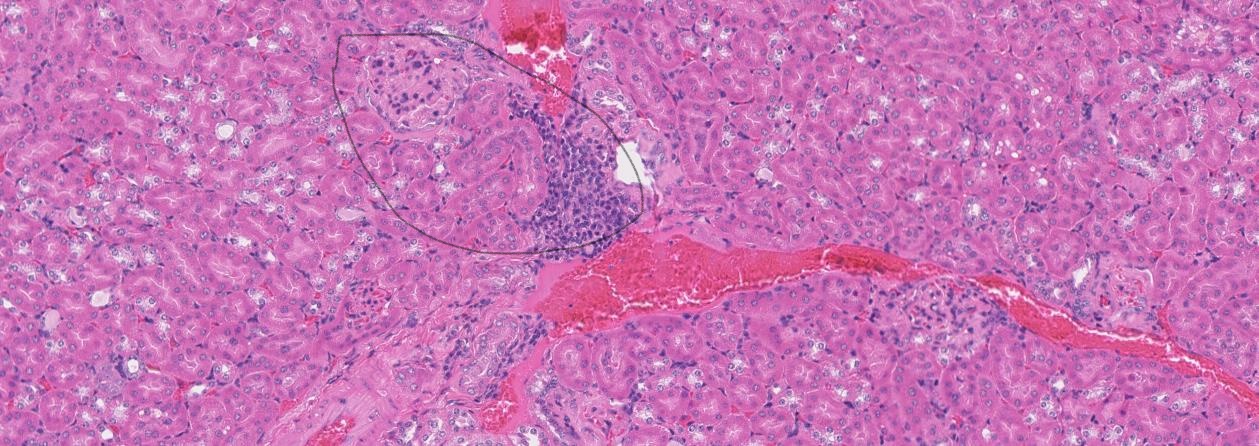


**B**


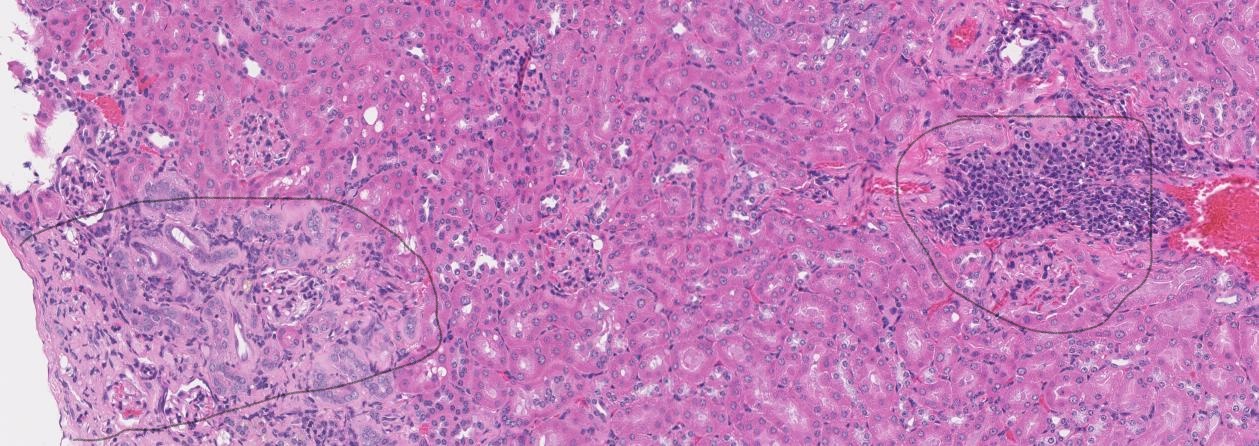


**Supplemental Figure 5. Representative images of kidney sections from old WT mice +/- rapamycin treatment.** (**A**) Kidney section from a 26 month-old C57Bl/6 mouse treated with oral rapamycin for 8 weeks showed less severe age-related lesions, including mild glomerulonephropathy and mild lymphoid aggregates (circled), than a kidney section from a placebo treated mouse (**B**), which showed moderate glomerulonephropathy and moderate lymphoid aggregates (circled on the right), and an infarcted area (circled on the left).

**Supplemental Figure 6. Analysis of age-related lesions in inbred and F1 hybrid mice.** Total composite lesion scores for C57BL/6Jnia and C57BL/6Jnia:Balb/cBy mice (8 per group) based on histopathologic analysis of liver, kidney, lungs, and heart. Values represent the mean ± SD., p<0.05 *, p<0.01 **, using a two-tailed Student’s t test.

**
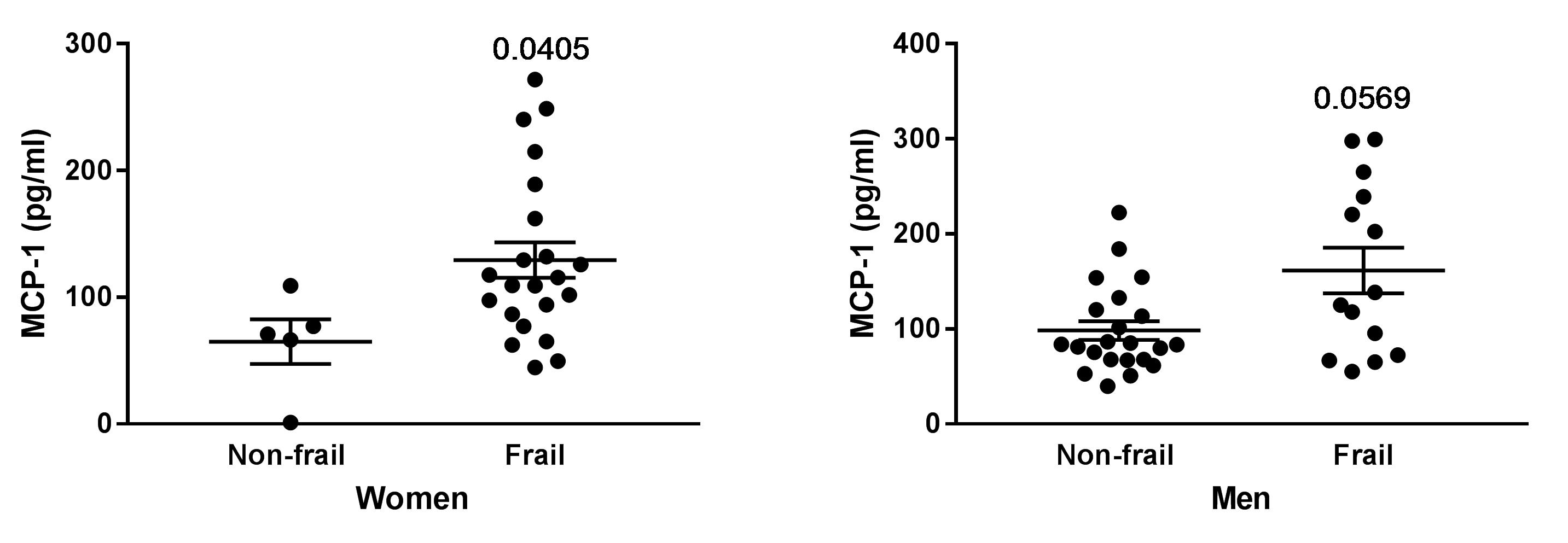
**

**Supplemental Figure 7. Stratification of MCP-1 concentration and frailty status by gender.** Graphed are individual values, the mean ± S.E.M, Mann-Whitney test.
