# Supplementary material for: Circulating levels of monocyte chemoattractant protein‐1 as a potential measure of biological age in mice and frailty in humans
Source: Aging Cell. 2017 Dec 31;17(2):e12706. doi: 10.1111/acel.12706 (PMC5847863; doi:10.1111/acel.12706)
Supplement: Supplementary file 2 [file ACEL-17-e12706-s002.docx]

**Supplemental Table 1.** Universal changes that occur with aging in humans and parallels in progeroid *Ercc1*^-/Δ^ mice.

| **Human age-related changes**  (Health & Medicine 2007) | ***Ercc1^-/Δ^* mouse age-related changes** | **References for mouse studies** |
| --- | --- | --- |
| 1. Brain / memory | - cognitive decline - cerebral atrophy - brain vacuolization | (Borgesius *et al.* 2011; Dolle *et al.* 2011; Harkema *et al.* 2016) |
| 1. Bones & joints | - osteoporosis - disc degeneration | (Vo *et al.* 2010; Chen *et al.* 2013) |
| 1. Eyes & ears | - loss of vision - loss of hearing - cataracts | (Spoor *et al.* 2012) |
| 1. Digestive & metabolic | - metabolic shift | (Karakasilioti *et al.* 2013) |
| 1. Urogenital | - renal tubule degeneration - urinary incontinence - testis atrophy | (Dolle *et al.* 2011; Tilstra *et al.* 2012) |
| 1. Dental | - ? |  |
| 1. Skin | - epidermal atrophy - alopecia - greying - loss of subcutaneous fat | (Weeda *et al.* 1997; Harkema *et al.* 2016) |
| 1. Function | - muscle wasting - ataxia - falls | (Gregg *et al.* 2011; Tilstra *et al.* 2012) |

Borgesius NZ, de Waard MC, van der Pluijm I, Omrani A, Zondag GC, van der Horst GT, Melton DW, Hoeijmakers JH, Jaarsma D, Elgersma Y (2011). Accelerated age-related cognitive decline and neurodegeneration, caused by deficient DNA repair. *J Neurosci*. **31**, 12543-12553.

Chen Q, Liu K, Robinson AR, Clauson CL, Blair HC, Robbins PD, Niedernhofer LJ, Ouyang H (2013). DNA damage drives accelerated bone aging via an NF-kappaB-dependent mechanism. *J Bone Miner Res*. **28**, 1214-1228.

Dolle ME, Kuiper RV, Roodbergen M, Robinson J, de Vlugt S, Wijnhoven SW, Beems RB, de la Fonteyne L, de With P, van der Pluijm I, Niedernhofer LJ, Hasty P, Vijg J, Hoeijmakers JH, van Steeg H (2011). Broad segmental progeroid changes in short-lived Ercc1(-/Delta7) mice. *Pathobiol Aging Age Relat Dis*. **1**.

Gregg SQ, Robinson AR, Niedernhofer LJ (2011). Physiological consequences of defects in ERCC1-XPF DNA repair endonuclease. *DNA Repair (Amst)*. **10**, 781-791.

Harkema L, Youssef SA, de Bruin A (2016). Pathology of Mouse Models of Accelerated Aging. *Vet Pathol*. **53**, 366-389.

Health NIo, Medicine FotNLo (2007). NIH Medline *Plus*. (aoa-r change, ed^eds): Friends of the National Library of Medicine, pp. 10-13.

Karakasilioti I, Kamileri I, Chatzinikolaou G, Kosteas T, Vergadi E, Robinson AR, Tsamardinos I, Rozgaja TA, Siakouli S, Tsatsanis C, Niedernhofer LJ, Garinis GA (2013). DNA damage triggers a chronic autoinflammatory response, leading to fat depletion in NER progeria. *Cell Metab*. **18**, 403-415.

Spoor M, Nagtegaal AP, Ridwan Y, Borgesius NZ, van Alphen B, van der Pluijm I, Hoeijmakers JH, Frens MA, Borst JG (2012). Accelerated loss of hearing and vision in the DNA-repair deficient Ercc1(delta/-) mouse. *Mech Ageing Dev*. **133**, 59-67.

Tilstra JS, Robinson AR, Wang J, Gregg SQ, Clauson CL, Reay DP, Nasto LA, St Croix CM, Usas A, Vo N, Huard J, Clemens PR, Stolz DB, Guttridge DC, Watkins SC, Garinis GA, Wang Y, Niedernhofer LJ, Robbins PD (2012). NF-kappaB inhibition delays DNA damage-induced senescence and aging in mice. *J Clin Invest*. **122**, 2601-2612.

Vo N, Seo HY, Robinson A, Sowa G, Bentley D, Taylor L, Studer R, Usas A, Huard J, Alber S, Watkins SC, Lee J, Coehlo P, Wang D, Loppini M, Robbins PD, Niedernhofer LJ, Kang J (2010). Accelerated aging of intervertebral discs in a mouse model of progeria. *J Orthop Res*. **28**, 1600-1607.

Weeda G, Donker I, de Wit J, Morreau H, Janssens R, Vissers CJ, Nigg A, van Steeg H, Bootsma D, Hoeijmakers JH (1997). Disruption of mouse ERCC1 results in a novel repair syndrome with growth failure, nuclear abnormalities and senescence. *Curr Biol*. **7**, 427-439.

**Supplemental Table 2.** Comparison of mouse models of progeria.

|  | ***Ercc1^-/Δ^*** | ***Bubr1^H/H^*** |
| --- | --- | --- |
| Mutated gene | *Ercc1* | *Bubr1* |
| Consequence of mutation | Compromised in multiple DNA repair pathways | Defective mitotic spindle assembly checkpoint |
| % normal protein expression | <10% | 10% |
| Organ systems affected: |  |  |
| Musculoskeletal | + | + |
| Dermatologic | + | + |
| Connective tissue (adipose) | + | + |
| Neurologic | + | + |
| Hematologic | + | - |
| Sensorineural | + | - |
| Immunologic | + | - |
| Cardiovascular | + | + |
| Other | Renal dysfunction, disc degeneration, infertility | Impaired wound healing, cachexia, facial dysmorphisms, infertility |
| Lifespan | Median: 4 months; | Median: 6 months |
|  | Maximum: 7.5 months |  |
| Premature cell senescence | + | + |

+/- indicates the presence of absence of involvement

**Supplemental Table 3.** qPCR primers

| Primer | Sequence |
| --- | --- |
| Cdkn1a (p21) Fwd | 5'-GCCTTAGCCCTCACTCTGTG-3' |
| Cdkn1a (p21) Rev | 5'-AGCTGGCCTTAGAGGTGACA-3' |
| *Cdkn2a* (p16) Fwd | 5'-CGTACCCCGATTCAGGTGAT-3' |
| *Cdkn2a* (p16) Rev | 5'-TTGAGCAGAAGAGCTGCTACGT-3' |
| *Mcp1* Fwd | 5'-GCATCCACGTGTTGGCTCA-3' |
| *Mcp1* Rev | 5'-CTCCAGCCTACTCATTGGGATCA-3' |
| *Gapdh* Fwd | 5'-AAGGTCATCCCAGAGCTGAA-3' |
| *Gapdh* Rev | 5'-CTGCTTCACCACCTTCTTGA-3' |

**Supplemental Table 4.** Study sample demographic characteristics stratified by frailty status.

|  | **Non-Frail (n=27)** | **Frail Subjects (n=36)** | **p-value** |
| --- | --- | --- | --- |
|  | **Mean (SD) or Number (%)** | |  |
| **Age (yrs)** | 79.0 (8.3) | 82.9 (5.8) | 0.046^1^ |
| **Male** | 22 (82%) | 14 (39%) | <0.001^2^ |
| **BMI** | 28.8 (2.8) | 31.0 (6.8) | 0.40^3^ |
| **Weight (kg)** | 85.5 (13.8) | 82.2 (19.3) | 0.45^1^ |
| **Height (cm)** | 172.0 (10.7) | 162.8 (9.8) | 0.001^1^ |

^1^Unpaired t-test,^2^Chi-square, ^3^Mann-Whitney

**Supplemental Table 5.** Linear regression relationships between MCP-1 and frailty score, adjusted for the indicated covariates.

| **Predictor Variables** | **β** | **Std. Error** | **95% CI** | **p-value** |
| --- | --- | --- | --- | --- |
| **MCP-1** | 0.86 | 0.25 | 0.36 – 1.35 | 0.001 |
| **MCP-1 + Age** | 0.90 | 0.23 | 0.43 – 1.36 | <0.001 |
| **MCP-1 + Sex** | 0.94 | 0.22 | 0.50 – 1.38 | <0.001 |
| **MCP-1 + Age + Sex** | 0.96 | 0.21 | 0.53 – 1.39 | <0.001 |

*MCP-1 values were natural log transformed to fit a more normal distribution.

**Supplemental Table 6.** Linear regression relationship between MCP-1 and frailty score among women and men.

| **Predictor Variables** | **β** | **Std. Error** | **95% CI** | **p-value** |
| --- | --- | --- | --- | --- |
| **MCP-1 in Women** | 0.74 | 0.24 | 0.26 – 1.23 | 0.004 |
| **MCP-1 in Men** | 1.45 | 0.43 | 0.57 – 2.33 | 0.002 |

*MCP-1 values were natural log transformed to fit a more normal distribution.
