# Supplementary material for: Circulating levels of monocyte chemoattractant protein‐1 as a potential measure of biological age in mice and frailty in humans
Source: Aging Cell. 2017 Dec 31;17(2):e12706. doi: 10.1111/acel.12706 (PMC5847863; doi:10.1111/acel.12706)
Supplement: Supplementary file 3 [file ACEL-17-e12706-s003.docx]

**Experimental Procedures**

*Mice.* Wild-type, *Ercc1*^−^*^/Δ^*^,^ and *p65*^+/-^;*Ercc1*^-/^*^Δ^* mice for this study were in an f1 C57Bl/6J and FVB background. *Ercc1*^−^*^/Δ^* mice were generated by crossing *Ercc1*^+/-^ mice in a C57Bl/6J with *Ercc1*^+/^*^Δ^* mice in a FVB/N genetic background. The f1 background reduces strain-specific pathology while still allowing for analysis of genetically identical animals. Mice were given a unique identifier by ear punch. Genomic DNA was isolated from ear tissue and the genotype of each animal was determined by TransnetYX (Cordova, TN) or as previously described (1). Wild-type (WT) f1 littermates were used as young normal controls. The *Bubr1^H/H^* mice and their WT controls were in a C57Bl/6 background as described (2). Serum was isolated from animals at the time of euthanasia (using CO_2_) by cardiac puncture.

*Generation and culture conditions of primary mouse embryonic fibroblasts (MEFs). Ercc1*^-/-^ primary MEFs were prepared from day 13 embryos derived from crossing inbred C57BL/6 mice heterozygous for an *Ercc1* null allele, as previously described (1). Cell lines simultaneously derived from wild-type (WT) littermate embryos were used as controls. Primary MEFs were cultured in a 1:1 mixture of Dulbecco’s modified Eagle’s medium and Ham’s F10 with 10% fetal bovine serum, non-essential amino acids and antibiotics, and incubated at 3% O_2_. Three independent MEF lines of each genotype were used.

*RNA isolation and qPCR.* Total RNA was isolated from MEFs using RNeasy isolation kit (Qiagen, Valencia, CA). Total RNA was quantified using a Nanodrop spectrophotometer (Thermo Fisher, Waltham, MA) and 1 μg of total RNA was used to generate cDNA with the Transcriptor First Strand cDNA synthesis kit (Roche, Basel Switzerland) according to the manufacturer’s specification. Gene expression changes in *Mcp1*, *p16* and *p21* were quantified by qPCR reactions using 20 μL reaction volumes using a StepOne thermocycler (Thermo Fisher, Waltham, MA) with input of 50 ng (*Gapdh*, *p21, Mcp1*) or 100 ng (*p16*) total RNA per reaction. Reactions were performed in duplicate for three separate experiments. Data was analyzed by ΔΔCt method and expression was normalized to *Gapdh*. qPCR primer sequences are listed in **Table S6**.

*Multiplex analysis of SASP factors.* Serum levels of MCP-1, IL-6, and TNFɑ were analyzed in a multiplex assay using the Milliplex Map Mouse Metabolic Hormone Magnetic Bead Panel kit (Millipore Sigma, St. Louis, MO). 10 μL of serum was analyzed in duplicate (n=9-11 per group) and analyte concentrations were quantified on a Luminex 200 (Luminex Corporation, Austin, TX) microplate reader.

*MCP-1 ELISA.* Serum concentrations of MCP-1 were surveyed for by mouse MCP-1 specific ELISA (Raybiotech, Norcross, GA). 50 μL of serum, obtained at the time of sacrifice, was used for ELISA per manufacturer’s specification and absorbance was quantified at 450 nm using a Spectramax i3 (Molecular Devices, Sunnyvale, CA) plate reader. All standards and samples were measured in duplicate (n=5-6 animals per group except *Bubr1^H/H^* and C57BL/6 controls where n=3 was used). 100 μL of conditioned media (48 h after splitting cells) from MEFs (n=3 per group) was used for the analysis of MCP-1 concentration by ELISA.

*Pharmacologic interventions.* Two year old C57BL/6J mice were obtained from the NIA Rodent Colony and treated with encapsulated rapamycin (Rapamycin Holdings, San Antonio TX) at 14 ppm for females and 42 ppm for males*,* or control (encapsulated only) diet for 8 weeks. Mice were sacrificed at 26 months and serum collected preceding necropsy. *Ercc1^-/Δ^* mice were treated with Dasatinib and Quercetin as described (3). Briefly, *Ercc1^-/Δ^* mice were treated weekly with a combination of Dasatinib (5 mg/kg) and Quercetin (50 mg/kg) starting at 4-6 weeks of age. Therapeutics were administered in 10% PEG400 by oral gavage. Litters with multiple *Ercc1^-/Δ^* mice were used to enable comparison of sibling pairs that were treated with therapeutic vs vehicle only. Mice (n=5-6 per group) were sacrificed at 16 weeks and serum collected by cardiac puncture.

*Kidney histology assessment.* Mouse tissues were collected at necropsy and placed in 10% buffered formalin for 48 hours, transferred to 70% alcohol, and subsequently processed into paraffin blocks for sectioning and hematoxylin and eosin staining. Histology slides were validated for age-related lesions by a veterinary pathologist and scored for lesion severity to create a composite lesion score for age-related renal pathology in each animal (4).

*Participants and research protocol.* Human procedures and the use of biospecimens were approved by the Mayo Clinic Institutional Review Board. A sample of 63 individuals (27 women and 36 men) age 65 years or older diagnosed with severe aortic stenosis and scheduled for surgical or transcatheter aortic valve replacement were recruited between July 2013 and May 2015 to the Mayo Clinic in Rochester, MN (5). Frailty assessment was conducted prior to surgery and was based upon the CHS criteria, defined by the following metrics: weak grip strength by electronic dynamometer (less than 17-21 kg for women and 29-32 kg for men, normalized to BMI), slow walk speed by a handheld ultrasonic monitor (less than 0.83 meters per second), self-report of low endurance and energy on the Center for Epidemiological Studies Depression Scale (self-report of exhaustion), unintentional weight loss (greater than or equal to 10 pounds in the prior year), and low physical activity by the Physical Activity Scale for the Elderly (men, less than 383 kcal expended per week; women, less than 270 kcal expended per week) (6). Fasted blood samples were collected in EDTA at the time of surgery and were centrifuged and stored at -80ºC. A Procartaplex Luminex immunoassay (Affymetrix eBioscience, San Diego, CA) was used for plasma MCP-1 quantification, according to manufacturer’s specifications.

*Cytokine and chemokines in human serum from the HANDLS study.* Serum inflammatory markers and cytokines (Figure S2) were quantified using Searchlight protein arrays from Aushon Biosystems (Billerica, MA) from a sub-cohort of participants from the Healthy Aging in Neighborhoods of Diversity across the Life Span (HANDLS) study of the National Institute on Aging Intramural Research Program. Participants provided written informed consent and the study is approved by the Institutional Review Board of the National Institute on Environmental Health Sciences, NIH. The coefficient of variance was calculated for the measured markers from a sub-cohort of 78 women. Details about this sub-cohort have been described previously (Noren Hooten et al., ATVB 2012).

*Statistical methods.* For mouse data analyses, unpaired t and one-way ANOVA tests were used to compare MCP-1 concentrations between sex, genotype, and drug intervention groups. Linear regression analysis was used to summarize the relationship between MCP-1 and age in wild-type mice. For human data analyses, unpaired t, Mann-Whitney, and Chi-square tests were used to compare demographic and anthropometric variables. Linear regression models were used to summarize relationships between MCP-1 and age, sex, and combined age and sex. Natural log transformations or non-parametric tests were applied for non-normally distributed variables as indicated. Analyses were performed using R 3.3.2, JMP Pro 10, and GraphPad Prism 7.

**References**

1. Ahmad A, Robinson AR, Duensing A, van Drunen E, Beverloo HB, Weisberg DB, Hasty P, Hoeijmakers JH, and Niedernhofer LJ. ERCC1-XPF endonuclease facilitates DNA double-strand break repair. *Mol Cell Biol.* 2008;28(16):5082-92.

2. Baker DJ, Jeganathan KB, Cameron JD, Thompson M, Juneja S, Kopecka A, Kumar R, Jenkins RB, de Groen PC, Roche P, et al. BubR1 insufficiency causes early onset of aging-associated phenotypes and infertility in mice. *Nat Genet.* 2004;36(7):744-9.

3. Zhu Y, Tchkonia T, Pirtskhalava T, Gower AC, Ding H, Giorgadze N, Palmer AK, Ikeno Y, Hubbard GB, Lenburg M, et al. The Achilles' heel of senescent cells: from transcriptome to senolytic drugs. *Aging cell.* 2015.

4. Ladiges W. Pathology assessment is necessary to validate translational endpoints in preclinical aging studies. *Pathobiol Aging Age Relat Dis.* 2016;6(31478.

5. Schafer MJ, Atkinson EJ, Vanderboom PM, Kotajarvi B, White TA, Moore MM, Bruce CJ, Greason KL, Suri RM, Khosla S, et al. Quantification of GDF11 and Myostatin in Human Aging and Cardiovascular Disease. *Cell Metab.* 2016;23(6):1207-15.

6. Fried LP, Tangen CM, Walston J, Newman AB, Hirsch C, Gottdiener J, Seeman T, Tracy R, Kop WJ, Burke G, et al. Frailty in older adults: evidence for a phenotype. *J Gerontol A Biol Sci Med Sci.* 2001;56(3):M146-56.

7. Ladiges W, Snyder JM, Wilkinson E, Imai DM, Snider T, Ge X, Ciol M, Pettan-Brewer C, Pillai SPS, Morton J, et al. A New Preclinical Paradigm for Testing Anti-Aging Therapeutics. *J Gerontol A Biol Sci Med Sci.* 2017;72(6):760-2.
